# Narrative analysis in health psychology: a guide for analysis

**DOI:** 10.1080/21642850.2018.1515017

**Published:** 2018-09-06

**Authors:** Gemma Wong, Mary Breheny

**Affiliations:** a School of Psychology, Massey University, Wellington, New Zealand; b School of Health Sciences, Massey University, Palmerston North, New Zealand

**Keywords:** Narrative analysis, narrative psychology, qualitative analysis

## Abstract

**Introduction:** Telling stories is a natural way to explain our experiences to others. Through telling stories, we come to understand these experiences, and to explain our own and other’s place in the world. Stories are an opportunity to present a version of ourselves and to shape how we would like to be seen. By analysing stories, we also reveal something about the social world beyond the immediate story. **Objectives:** Applying a narrative approach to interviews can be challenging for the beginning narrative scholar. This paper provides a worked example of a narrative psychology approach to analysis. We present examples identifying the different levels at work in stories: personal stories, interpersonal accounts, and social narratives. Beyond identifying the levels, we offer further suggestions to assist with the narrative analysis of interview transcripts. These suggestions provide a way to start to understand why stories have been told, and what their telling reveals more broadly. To illustrate how to build a narrative analysis, we use a small set of interviews with older people in residential care.

Articles arguing for a narrative psychology approach often conclude by stating that detailed analysis of narrative can aid understanding of identity and social life. Empirical papers seldom include much detail of how the analysis was conducted, moving from the participant recruitment to the findings as if the production of the analysis itself were self-evident. In this paper, we provide a detailed worked example to help the beginning scholar produce a narrative analysis from interview data. Examples are provided that demonstrate the levels of analysis, and the ways that characters, settings and rhetorical features can be used to understand the stories people tell. This paper demonstrates how to build an analysis from data collected for a narrative psychology project and aims to provide flexible ways to think about and engage confidently with narrative data.

## What is a narrative approach?

Narrative research is not a singular approach; rather, it refers to a range ways of examining the role of storytelling in understanding identity and social life (Riessman, 2008). Narrative approaches recognise that the stories people tell are significant; they are worthy of close examination, both for the ways they signal the important events of people’s lives and how they reflect and reinforce social identities. Narrative analysis is concerned with stories as a whole rather than separating them into themes or abstracting them into discourses (Murray, 2003; Riessman, 2008). Because people make sense of their experiences through storytelling, it is this storying of experiences that is the focus of analysis within a narrative approach.

Telling a story or thinking in stories comes automatically to most people because people have been socialised through stories their whole lives. By listening to cultural stories and children’s stories, hearing people tell us about the happenings of their day, and watching plays and movies, we absorb narrative structure. Because of this, we use stories to put events into an order that makes sense to us. Stories are useful to tell others what events happened, to explain why those events are important, and to understand what they might mean for who we are (Somers, 1994). Stories are not just a set of events ordered in time, they are a set of events put into order to convey a particular meaning, to provide an explanation of who we are and how others should see us (Riessman, 2008).

Social and cultural familiarity with storytelling provides the building blocks that we use to shape the events of our lives into stories. These building blocks are of two types. The first is the structures that tell us how to set up a story, how to describe the scene, how to order the events and actions, and the skills to move the audience to the resolution of the story. Secondly, stories are built on taken for granted understandings of how social life does and should work (Murray, 2018). Because of this, the stories people tell give us insights into the, often unspoken, rules for reacting to and interacting with the world, because they reflect broader narratives of social life that we have absorbed (Crossley, 2008). Because these are part of how we assume the world to be, they are hard to see, but vital for analysing why some stories are told and not others. Michael Murray describes this by saying we ‘swim in a sea of stories that seeps into our consciousness and our very identity’ (2003, p. 98). A narrative approach aims to make visible that which has already shaped our consciousness and identity.

## What is a narrative?

There are a variety of distinctions drawn in narrative theorising between a story and a narrative. For the purposes of this paper, we make the distinction that a story is the account of events the speaker tells, while a narrative refers to the wider accounts of social life that are drawn upon to tell a story. Consequently, we suggest that people tell stories, and that they use widely available narratives to tell these stories. By using the word narrative, we shift to the analysis of a story, the ways the analyst comes to understand not just what the story says, but what it might mean and how it might be linked to other stories. Although people tell stories that are personal to them, the structures that shape these stories do not belong to them alone (Riessman, 2008). Even profoundly personal stories are embedded in the social, cultural, and material conditions which make certain stories and identities available and others unavailable (Silver, 2013). Narratives are the overarching structures and understandings that influence how and why these stories are told. This is what narrative psychology is concerned with: ‘the structure, content, and function of the stories that we tell each other and ourselves in social interaction’ (Murray, 2003, p. 95).

There is no definitive approach to narrative analysis (Polkinghorne, 1995). Different authors have analysed narratives in different ways, and the ideas they offer can be used flexibly to analyse data. Narrative researchers have distinguished ‘big stories’, the highly structured narratives of past events, from ‘small stories’, the everyday interactional stories people tell in ordinary conversational encounters (Bamberg & Georgakopoulou, 2008). The distinctions between the highly rehearsed and structured ‘big stories’ and the fragmented and incomplete ‘small stories’ have been used to propose different approaches to analysis. Wilson and Stapleton (2010) argue that such distinctions are not always analytically useful. Using the levels of analysis approach demonstrated here, both highly structured narratives and everyday interactional accounts can be analysed in similar ways. They can both be understood as a specific account of particular events, located within an interactional setting, and as reflecting broader understanding of social life.

## Identifying a story

There are several ways to identify a story. In a story, events are typically described as part of an evolving plot line: first this happened, followed by this, and subsequently, that. To make these events sensible over time, most stories use characters performing some kind of action brought together in a plot line (Grbich, 2013). For example, speakers regularly use days and times, places and character descriptions to signal the beginning of a story. Sometimes people tell straightforward stories, with the scene set beautifully, and the characters colourfully described. These resemble a traditional ‘story’: a clear sequence of beginning, middle, end; characters, setting, plot, and conclusion, presented in an unbroken sequence like a carefully rehearsed anecdote. Not all stories move so seamlessly or have a clear structure. Some stories are complicated, overlapping and unfinished, with digressions and sub-plots. Intuitively we understand that digressions are introduced when details are understood as necessary for the audience to understand the message of the story. Lack of clear narrative structure can be analytically important. When stories are started, abandoned, and re-introduced, it is valuable to examine why this occurred. Restricting analysis to accounts that meet all the structural requirements of a formulaic story might neglect particularly poignant or tense stories that participants struggle to finish. The narrative quality of these accounts of everyday life may be less immediately obvious, as they do not necessarily follow the structure we have been socialised to expect from a story. Some participants provide much less detailed stories overall (Gubrium & Holstein, 2012). It would be easy to dismiss the interviews with less detailed stories as not ‘good’ narrative data, but the brief and often bald accounts of experience with little embroidering are equally informative of social life.

## Demonstrating narrative analysis: animal therapy in residential aged care

To demonstrate how to build a narrative analysis, we use a set of interviews with older people in residential care regarding their experience of involvement with an animal therapy programme (Wong, 2016). The research received ethical approval from the Massey University Human Ethics Committee. The data includes both some well- crafted stories and some brief stories that were analytically important. This data set was chosen to illustrate the analytic process; consequently, the results of this study are not presented in detail, but are available elsewhere (Wong, 2016).

## Levels of narrative

Identifying narratives at work in the interview data requires analytic distance from the stories that people tell. This can be difficult as narrators are adept at constructing a story that draws the listener in and makes their account of the world seem sensible. Different approaches to narrative analysis have focused on different aspects as ways to gain analytical distance. Riessman (2008) describes different approaches that identify thematic similarity in the stories told, or attend to structural features of stories, or examine stories as performing interactional identities. Rather than concentrating on one of these aspects, Murray (2000) and other narrative researchers (Phibbs, 2008; Somers, 1994) suggest looking at the different levels stories are simultaneously told at, as a way to interpret the stories participants provide.

A story functions at multiple intersecting levels at the same time. By separating out these levels, analysts can better understand the ways that personal stories are situated within wider narrative structures available in society. Murray (2000) describes four levels of narratives: personal, interpersonal, positional and ideological. These levels are not separate, as ideological narratives provide the positions available for speakers that play out in interpersonal interactions. In practice it can be difficult for beginning researchers to distinguish between interpersonal or positional levels, and recognise how this reflects ideological narratives. Here we present a simplified structure of three levels as a straightforward way to start analysis. We make distinctions between the ways these three levels function: personal stories, interpersonal co-creation of accounts, and social narratives (Stephens & Breheny, 2013). This structure provides a framework for thinking about each level in turn. Separating out the levels is not the end of the analytic process. Narrative researchers are typically interested in how at least some of these levels work together (Stephens & Breheny, 2013) as well as how this contributes to a wider narrative analysis of the data set. By using worked examples to illustrate the analysis of each of these levels, we aim to make explicit the choices that underpin a narrative analysis and show the interactional and constructive nature of analysis.

## Personal stories

The individual story is the immediate focus in narrative analysis since the stories told about everyday life are the basis of narrative psychology. These are the accounts of events or experiences that people provide to others, which order events in time and typically provide some sense of causal linking of events (Murray, 2003; Polkinghorne, 1995). Stories allow people to provide their own context; when people tell their story they give the details that are important to understand the message of the story. Stories can be used to reframe chaotic experiences in a predictable narrative order, or to highlight or resolve tensions. In this way, the unexpected can be made sensible and predictable.

To illustrate the personal story level of analysis, below is an extract in which Yvonne describes how she came to live in residential care. She stories profound transitions, her increasing immobility due to her Parkinson’s disease, and the death of her husband in three sentences to story this transition.

Gemma: Can you tell me, Yvonne, how you came to be in here?

Yvonne: I got Parkinson’s, so I couldn’t move or do anything. So, my daughters, my husband, thought it was better for me, to be here. My husband died, that was all.

Gemma: I’m sorry to hear that. Excuse me, I’ll just shut that door. And how do you feel about being in here?

Yvonne: I know I can’t look after myself, so I just have to be here. [Gemma: Sure.]

Interviews vary in the extent of storytelling provided; some participants readily tell detailed stories, whereas others provide much simpler stories as Yvonne does. Although brief, this illustrates a whole story of Yvonne’s transition to residential care, with a beginning, middle, and end; characters who play roles in the narrative and a resolution. In this way, Yvonne stories a simple account that reframes residential aged care as an inevitable outcome given her circumstances. In spite of widespread narratives of ageing as decline, many older people do not anticipate needing residential care. In this way, Yvonne’s shift to residential care troubles a trajectory in which she may have expected to age at home. Stories do not reveal to others what happened or who we are in any transparent way, but are a process of telling others how we would like to be seen. Even in this simple account, Yvonne presents herself as logical, able to comprehend her situation and recognise the impact of her declining health on others, and to conclude that residential care is the sensible alternative, restoring coherence to Yvonne’s altered living situation.

## Interpersonal: story telling

Stories are told to an audience of at least one other, so they are a joint project with an active audience (Mishler, 1986; Murray, 2018). Because of this, stories are shaped to fit the expectations of the interactional moment. In the context of an interview, participants may orient to telling a good and entertaining or insightful story. Andrew illustrates this explicitly by comparing his humorous approach to other participants’ more reserved accounts:

Andrew: Well I gotta tell you something, in this place it’s quite funny. Because I’ve often thought of writing a book about it, you know, ‘My Life Inside and Out’, you know. And, you know, some are very snooty, have you noticed that?

Gemma: And just, I think sometimes people don’t know what to say, so they didn’t say much more than sort of ‘yes’ or ‘no’, whereas it’s quite good asking you a question and you’ll tell me a story, whereas not everyone-

Andrew: [Interposing] I like telling all the stories, you know. [Gemma.: Yeah, it’s really good [laughter].] [Laughter] I’m full of little stories.

Andrew, like many participants, views his role as telling good stories and sees these as both enjoyable and useful for the researcher. Because story recipients take an active part in shaping the story that is told, the story is situated in this particular interaction, shaped by the responses the interviewer gives (De Fina & Georgakopoulou, 2008). Andrew also explicitly uses humour to draw Gemma in, encouraging her to characterise the other residents as haughty in contrast to his jovial and engaging approach. This illustrates the way that interview participants work to situate the interviewer in different ways: for example as a confidant, as a co-conspirator, as an empathic listener. Identifying these roles can aid the interpersonal level of analysis. While the immediate listener is usually the interviewer, the person telling the story also has an awareness of the ‘unknown’ audience (particularly if the interview is being audio recorded). For example, other researchers, supervisors, thesis readers and journal readers may all be part of this unseen audience. The awareness that their story will reach beyond the moment of the interview influences what the participant tells.

The characteristics of the researcher and the relationship between the participant and researcher also influences both the story and how it is told (Silver, 2013). The following illustrates how the interviewer, Gemma, in her late twenties, plays a role in shaping what is said:

Valerie: Well, only the last, it sounds funny to you when I say ‘only the last 20 or 30 years’, which is a small part of my life, but I lived in most other cities.

Valerie begins to discuss the recent events of her life, but then corrects herself as she realises her recent history would encompass all of Gemma’s lifetime. In addition, because Valerie knows that Gemma has an academic interest in ‘older people’, she may be primed to tell her stories through a lens that emphasises this part of her identity. In this research, the participants may be more likely to describe their satisfaction with the animal therapy programme and the care facility to Gemma, the young health researcher, than if they were asked by an older friend or another resident. Examining the stories as an interpersonal co-construction means recognising the story is a local rendering of these events for this particular audience.

The interviewer also supports and encourages some versions of experience more than other accounts. This is particularly the case when stories challenge the interviewer and the overriding narrative that they hold about the experience they are researching. In the following personal story about the experience of animal therapy, Jean tells Gemma a story about enjoying the animals for the physical affection they bring to her life in residential care. In response to this simple account, Gemma encourages Jean to tell a story about how she came to live in residential care, a standard technique to encourage participants to story their experiences. In response, Jean laughs and says, ‘I’m old, I’m bed-ridden mainly’ and reasserts that the animals are just a simple pleasure in her life. Gemma again tries to overlay a narrative of choice and ‘liking’ living in residential care, which Jean again resists. For Jean, being old and bed-ridden is not about choosing where one lives or ‘liking’ where one lives, it is about being well looked after. To reinforce this, Jean tells a parallel story of her cat who also lives in its own residential care facility – a cattery in another city. In this case too, the participant notes that her cat is the boss of the place and well cared for. The following extract demonstrates how the story plays out between the young interviewer and the older participant:

Gemma: Oh, ok. Well … they call the programme ‘Animal Therapy’, have you ever heard of that before?

Jean: Yes I have.

Gemma: What do you think about that?

Jean: I think it’s a marvellous idea. And I think particularly, someone like me who’s been bed-ridden for a while, someone bringing a puppy or a kitten and letting them snuggle up against you is lovely.

Gemma: Can you tell me about how you came to be in here?

Jean: In bed like this?

Gemma: Yes, or in the rest home or both.

Jean: I’m just old [laughter]. No I um, I don’t know how to describe it really, I’m old, I’m bed ridden mainly, people come in with their wee kittens or their dogs and we have a wee cuddle together. That’s about it.

Gemma: Were you living at home before you moved in here?

Jean: Um, not really in a rest home. I don’t really know how to describe that. I’ve always been looked after in some way. I don’t know. [Gemma: That’s fine.] I’m really old, I don’t remember. (…)

Gemma: Do you quite like living here?

Jean: I don’t have much choice actually. But no, they’re very good to me and look after me. No I’m very fortunate.

Gemma: And do you ever see the, there is a cat that lives here as well isn’t there?

Jean: The cat that belongs to me lives at Masterton in the cattery there, which is a very good cattery, it’s well looked after. In fact she’s the boss cat there as far as I can make out. Locks the other cats into place.

Gemma: Oh I see. Ok, great. Is there anything else you’d like to talk about, about the animals or the programme?

Jean: No I, I just like to see them. Particularly the young ones the puppies or the kittens. And they’re little cuddlers, you know. [Gemma: So, it’s the affection that you like?] Mm [nodding] (…)

Gemma: Mm. Alright, well thank you so much for your time today.

Jean: Oh that’s – I’m always glad to talk about animals.

Gemma: Yeah, can I get you a cup of tea or anything?

Jean: No thank you, I’m so well-looked-after.

Gemma: Oh good, I’m glad to hear that. What have you got planned for the rest of your day? Do you have a lot planned for the rest of your day?

Jean: No. [Laughter] You don’t have plans when you lie in bed all day.

Gemma: Do you- you don’t get to leave at all?

Jean: Um, not really, no. I don’t want to. I just like being here, and they’re so good, they really do look after you.

Gemma: Excellent, well alright, I hope you have a lovely rest of your day.

The mismatch between Gemma and Jean is best illustrated when Gemma asks Jean what she has planned for the rest of the day. This provokes laughter as Jean explains that making plans is not part of life when you are bed-ridden in residential care. Gemma responds with a level of bewilderment with ‘Do you- you don’t get to leave at all?’, introducing an alternative narrative of a good life as based upon choices, plans, and engagement with a wider world. From Gemma’s perspective, residential care is limited and restrictive. Jean instead returns to the message of her story, that she is well cared for, and reinforces this message by responding to Gemma’s offer of a cup of tea with the decisive ‘No thank you, I’m so well-looked-after’. Jean’s account of a good life fundamentally differs from Gemma’s; it is about physical affection, good quality care and needs met reliably. Finally, in recognition, of Gemma’s youthful lack of comprehension, Jean reframes her experience of residential care in ways that she feels that Gemma can understand. In contrast to her previous account, she states simply that she does not want to leave, she likes being there, and restates that she feels well cared for. By analysing the story as a joint construction between Gemma and Jean, we can see the completely different narratives they use to understand what it means to have a good life, which structures their disparate assessment of life in residential care. The story that Jean tells does not belong to her alone, but is a co-construction between them both. It is worth noting that this mismatch was not apparent to Gemma during the interview, as the participant and interviewer are carried along by the story during the interaction.

## Social narratives

In doing narrative research, we are interested in more than the personal stories told to an audience. We are also interested in understanding how these stories reflect society more broadly. In narrative theorising, Somers (1994) calls these culturally shared stories ‘public narratives’. Murray refers to them as ‘ideological narratives’. In essence, these all refer to the understandings of ‘how things should be’ that are shared by society at large: shared understandings of social life that shape what we believe to be good, shape how we believe people should act, and how we can understand why people do what they do. Understanding the social narratives that shape the stories people tell requires the analyst to reflect on the assumptions they and the participant might share about the world. This includes examining the way the story is supposed to go (e.g. good triumphs over evil, or adversity is overcome). It also includes examining what the characters are entitled to do and why they have that right. Examining these shared understandings in the stories people tell reveals the society the analyst and the participant both live in*.* For example, old age as a state of decline is a shared understanding that was often used by participants in residential care, either as a narrative they used to contrast their own experience (‘I’m not old in that way’) or to explain their experience (‘of course this is what my life is like, because that’s what being old is’). All topics have narratives that shape the expected course of events, and interview participants and interviewers both reflect, reinforce and resist these in their accounts.

To illustrate the social narratives that shape the personal stories, we present an extended account from Cathy describing her limited interest and involvement in the animal therapy programme. She explains this as due to her ability to maintain mobility outside the home in comparison to other residents, such as Annette, whose limitations she hints at before trailing off. Cathy signals the beginning of her personal story by saying ‘Sunday’s an example where I called a taxi … ’. This orients the reader to a particular day and time through which Cathy stories herself as ageing actively out in the community. Cathy recounts a Sunday walk to the waterfront as a pleasant alternative when her usual family outing is cancelled. In this way, Cathy uses social narratives of active ageing to situate herself as an older person, still physically active, socially interested and interesting, and located in the world outside residential care.

Cathy: Ah [pause] I can’t imagine what could be done um [to improve animal therapy], because prolonging the meetings – and because I mean in that short time we get the history, if she has it, on the animal and … and I honestly can’t think of any way, and no I don’t think that a more formal approach is the answer. Now Annette might have some–and she … For example, I’m still managing to be mobile, ah [pause] growing less mobile, growing more tired all the time. Nevertheless, I mean, Sunday’s an example where I call a taxi and ah Sunday I–I usually see my family but I wasn’t–they weren’t able to see me on Sunday because you know Christmas and social engagements and they were invited to a barbeque, and–but I wasn’t going to spend the whole lovely day, because Sunday was lovely [Crosstalk] [Gemma: Beautiful.] And um taxi down to AquaCentre and then, delightful walk as far as the rotunda ah, and, three really pleasant social encounters, so I mean that was what one gets to long for in here, ah to meet someone like you for example, that I can talk to you. Because I don’t actually get that much chance to talk, so that’s why you find people like me pretty gabby [Gemma: Laugh] [Cathy: Laugh] And, yeah. And, uh, so Sunday was for me a very successful day except that at the end when I went back to AquaCentre where they’re always kind enough to ring me a taxi, I encountered something I’m beginning to encounter a little bit more, which was that a taxi came, saw me, and did a U turn [Crosstalk] [Gemma: Really?] Because what am I, old woman, with a walker that has to be put in the car, probably going to some nearby rest home. Ah, we’re not popular. And I have been refused, ah, ‘No I’m sorry I’m not free I have to get my notes done’. That sort of thing.

Gemma: That’s terrible, I’m sorry to hear that.

Cathy: Yes, has to do entirely I think with the Total Mobility–with age, Total Mobility with age where they only get half from me, and then they have to wait and (…) you know in other words they don’t get the full fare to begin with [Crosstalk] [Gemma: Up front] they get half of it, and then they send their bills in and Total Mobility send them the rest of it [Crosstalk] [Gemma: That’s terrible.] So I had to walk all the way then from AquaCentre, which I wasn’t expecting. I had pretty well used up most of the energy but I said, well, make it a positive thing in your life today, you’ve got an extra-long walk. So I just took my time and dawdled along to City New World and got ah, got in a taxi that was there. So, Gemma, it’s been a bit of a lost effort for you.

This account of active ageing is troubled by a detailed sub-story of Cathy’s difficulty in getting a taxi, and her interpretation of why that happened. The taxi driver’s refusal to accept the fare when he sees that Cathy is an older person with a walker forces Cathy to see herself through this lens, as physically and financially troublesome: requiring help with her walker and paperwork to claim the full fare. Instead of ageing actively, she is positioned as burdensome and declining. This works to trouble the distinction her story set out to make, that Cathy is different from those other older people who are immobile and unable to engage with the outside world. The way Cathy describes what she thinks the taxi driver sees when he looks at her tells us about how Cathy is positioned by a narrative of ageing as decline and burden, how her body reads as aged to others, and the effect she sees that having on her everyday life. Telling stories is an embodied process (Polkinghorne, 1995). What we are able to say depends upon the physical condition of our bodies and the social expectations that brings, for example, as able bodied or disabled, young or old (Holstein, Parks, & Waymack, 2011). The kind of body a person has is deeply embedded in their story, because their body can be the topic, the cause, and the instrument of their story (Becker, 1997; Frank, 1991, 1995). Cathy returns to her initial message by storying an extra-long walk as a positive outcome, repairing the apparently conflicting sub-story of the difficulty taking a taxi. In this way, she reframes this unexpected ending as part of a narrative of active ageing which also includes a relentlessly positive attitude (Breheny & Stephens, 2019). Her final sentence returns the listener to the original story, that interviewing Cathy is a lost effort for Gemma, as she is not old in the same way as the residents that animal therapy is designed to benefit, residents like Annette.

These social narratives are not contained; they often have elements of alternative accounts within them. The way the story is resolved shows the relative merit the storyteller places on alternative narratives available to account for their circumstances. For example, a narrative of ‘active ageing’ might recognise decline as inevitable, whilst also framing decline as something to resist. Cathy points to this with ‘I’m still managing to be mobile, ah [pause] growing less mobile, growing more tired all the time’. Some narratives fit together compatibly, while others create tensions, which the narrator must work rhetorically to resolve (Breheny & Stephens, 2011). The ways that these stories are resolved show the relative power of the available narratives.

Historical changes can also alter wider narratives of social life, because what can be storied (i.e. what makes sense), changes over time (Bell, 1999)**.** For example, narratives of ageing as a state of decline and disengagement have given way to expectations for people to age ‘actively’ (Breheny & Stephens, 2019). Expectations for active ageing can mean living in residential care represents (to society and to the older person themselves) a failure to maintain health and vitality into old age. Social expectations are constantly shifting and these changes influences what can be told. The way an experience is storied at one time will be different from an account given at a different time.

## Analytic devices

The previous section outlined different levels a story is simultaneously told at. The next section details further analytic tools that may assist in building an analysis from the data, illustrated by the research in residential aged care. These are ways to identify the stories told, to begin to understand why they have been told, and what their telling reveals about wider narratives. Considering when, where and by whom such stories can be comfortably told, as well as when, where and by whom such storytelling would defy social convention, leads the analyst into a more nuanced understanding of the narratives that structure social life.

### Structural features

Narrative researchers have identified stories as having typical structural forms and these structural features can help the analyst to begin to see the story as a construction to serve an interactional purpose. For example, Labov’s (1997) identification of the typical structural features of a personal story (abstract, orientation, complicating action, evaluation, resolution, and coda) provides a way to understand the speaker’s explicit purpose in telling the story. In the example from Yvonne on page 7, the features of resolution and coda particularly demonstrate how Yvonne shifts from the events described in the past tense, to the present tense to both resolve the events described and return the story to the present. She does this to conclude: ‘so I just have to be here’. A structural analysis of this account highlights the inevitability of residential care, a resolution shared across many of the interviews for this research.

Alternatively, identifying the ways that stories adhere to broad genres such as comic or tragic, heroic or romantic may be a starting point for analysis. These broad narrative structures situate experiences in familiar plot sequences that listeners can recognise and consequently link the events to. By questioning how the speaker constructed their account, or linked specific events to a familiar genre, we can begin to understand why they shaped it in that way (Grbich, 2013).

### Stage and setting

Stories are always set in a particular place and told from a particular setting. These may not match – for example, Cathy’s story takes place at the waterfront, but is told from the setting of the lounge of a residential aged care facility. Both the waterfront and the lounge are physical, social, and psychological settings. Both the location of telling and the location of the story itself shape the story and the unfolding events (van Vuuren & Westerhof, 2015). Because these contexts shape the story, they need to be explicitly considered in the analysis (Levitt, Motulsky, Wertz, Morrow, & Ponterotto, 2017). For example, the residential care setting was a key part of all the participants’ stories in the animal therapy research, whether it seemed immediately relevant to the question about animals or not. We can see this in Andrew’s transcript below, when the story told to Gemma quickly veers away from animal therapy directly. The fact that it does, and that this happened repeatedly through Andrew’s interview, underscored the lack of impact the animals had in the wider context of what it meant to Andrew to live in residential care. Andrew used the questions about animals as an opportunity to lead into stories that moved the listener away from the animal therapy programme and the care setting, by setting his stories in a different time as well as a different place. He refocused attention on animals he had owned previously, his technology skills, extensive travel, and the interests of his life before entering residential care.

Gemma: Does that mean you prefer when dogs come in here, than cats? Or do you not mind what animal Stephen brings in?

Andrew: I don’t mind cats. I had a cat. Long time ago. But it got run over. [Gemma: Oh, sorry to hear that.] They were a bigger part of me than the family. [Gemma: Really?] [Nods].

Gemma: Can you tell me more about that?

Andrew: Just never got on- they played sports, mad on sports, and I was mad on art, I was mad on, you know, music. I-I don’t care. I mean, as far as I’m concerned I’ve led a pretty full life. I had photography, and what you see in here now is nothing [indicates VCR tapes and recording equipment]. I had about 50 times the amount of stuff which I’ve got here. The place I had, I had TV sets galore, all round the place, above my head and god knows what, I was cutting movies. The only thing is the sad part about it is I couldn’t bring the rest of my cassettes, because I’ve got some terrific recordings. And some of the BBC hard talk and all that stuff, you know.

Gemma: Why couldn’t you bring them in?

Andrew: It takes about half the space of this room. I’m talking five thousand!

The stage for Andrew’s stories illustrates the separation of his ‘full’ life outside the residential care facility from the everyday experiences in the facility. He stories his identity to paint a picture of himself as a worldly and relevant man, technologically adept and conversant with current affairs. Analysing the story both for where it does take place and for where it does not, as well as considering the location of the participant and the interviewer is a key analytic tool. The places that stories are recounted shape the rights and responsibilities of different speakers. In this research, residents have different rights to speak than staff in residential settings, and both staff and residents differ from volunteer service providers. Consequently, place is different for each of the characters in this setting: for the residents it is a not-quite-home, for staff a place of work and professional identity, for volunteers a place of community service.

### Characters

The setting of the story has implications for how each of the characters in the story are described as having particular roles and responsibilities. People perform their identity through telling stories (Riessman, 2008). Socially available narratives make a range of identity possibilities available, from broad identity categories based on age or gender, through to explicitly moralised identities such as having a ‘good life’. In this way, people tell stories to claim or avoid a particular identity (Bruner, 1990; Sarbin, 1986). Available narratives come with possible characters, whether this refers to generic characters such as the hero or the villain, or whether it refers to more specific possibilities such as the loving daughter or the devoted mother. In the data presented here, the participants variously presented themselves as loved and loving family members, as active, engaged and engaging older people, as appreciative care recipients. Each of these characters has a function in the story, which influences *why* the character has been described in that way, and what effect the listener has had on the character description. The personal, interpersonal and social levels of analysis work together to make some characters available and others unavailable. Narrative characters provide a way to represent a positive identity without having to make claims directly. Similarly, they provide an indirect or subtle way to question the motives of other people, particularly close family members or those in power relationships such as health care professionals. Whether presenting ourselves positively or others in a less flattering light, telling a story can lead the listener to a conclusion that may not be easily stated outright.

### Tension in the story

#### Storying discomfort

Narratives are functional and purposeful (Riessman, 2008), and choosing to convey a message through storytelling may indicate that it is difficult for the speaker to present their message in other ways. Sometimes stories seem incomplete because speakers hesitate to deliver the punchline in an explicit way. Instead, the speaker stories events, which lead the listener to a conclusion without having to be explicit. This demonstrates how the interpersonal level relies on a shared understanding from the societal level: both speaker and listener implicitly understand what the story should be, and therefore can tell when it deviates, or fill in the ending if the story trails off. This applies both to the immediate listener, and to how a broader audience might hear the story. Audiences understand the unspoken message because the assumptions upon which narratives are built are shared.

The way a message is conveyed without being explicitly stated is illustrated in Valerie’s story about why she lives in residential care. The following extract comes at the end of a meandering discussion of whether or not Valerie had pets before she moved into residential care. To contextualise this, Gemma asks how long Valerie has been in residential care and whether Valerie likes living there. Valerie never directly answers whether she likes living there, but uses this moment to explain why she moved into residential care, and deliver the message to the listener that she’s grateful to be looked after but the situation is not necessarily something she would have chosen for herself if there were other options.

Gemma: And, do you like living here?

Valerie: I’m being very well looked after and I’m very fortunate because ah, I was at, ah home, um, and my husband had been dead some time, and I was on my own, and they lived in Halswell [close by] but not with me or me with them, and when they found the house, I’ll say ‘we’re in’, I’m not in it but um when I came in here and my part of the house, um my granddaughter’s in it now, so it’s nice I just left it as it was and all the furniture and crockery and everything’s there. [Gemma: That’s handy.] Mm, so that’s been worked out very well.

Gemma: And what was it that made you move in here?

Valerie: Ah, well I came, my daughter was going away for what they call respite, for respite care, and that was respite from me, and I went into a place still, not in Halswell, for the fortnight she and her husband went away, always thinking I would be out and in it again, but by this time it was obvious that I’d have to spend time here and I suppose it grew from that into full time. And I know it’s right. And she still, my daughter that was here this morning, she just lives round the block. [Gemma: Oh that’s handy.] She comes many days, she works. So it’s worked out very fortunately for me.

Valerie’s story is located within an overarching narrative of the family and family care of older people as preferable. To story her response to Gemma’s question, Valerie draws on the character of the ‘dutiful daughter’, who could no longer continue to support Valerie, who visits often even though she works. By indicating that her daughter needed respite from Valerie, she points to a tension in the story without directly criticising her daughter. Valerie casts herself as the grateful older person who recognises that her daughter was seeking respite from caring for her. Gemma affirms Valerie’s daughter living nearby as positive and appropriate indicating their shared understanding of the narrative of family life. The new house for the family does not include Valerie, she is not part of the ‘we’ that is accommodated within ‘when they found the house, I’ll say ‘we’re in’, I’m not in it but um’. Valerie is both included and excluded from the ‘we’ that she refers to and her account stories her appreciation of being considered whilst acknowledging her limited agency in deciding the arrangements. The resolution of the story draws together two key phrases from the account into the final sentence ‘So it’s worked out very fortunately for me’. This brings together Valerie’s claim that she is fortunate with her shifting reference to the situation working out. Earlier it was described as ‘been worked out’, where Valerie’s agency in the situation is unclear.

#### Humour

Examining when and why humour is used in a story can be another useful device. Tensions can be revealed and resolved with humour, and uncomfortable topics can be raised. For example, in the extract used to illustrate the interpersonal level, Jean uses laughter to show that she is aware of her limitations, and to diffuse the possible awkwardness of needing to explain the mismatch in understanding when Gemma repeatedly responds with confusion. Cathy also uses humour with self-deprecation to simultaneously show that the residential aged care facility can be a lonely place for her, and to acknowledge the negative stereotype of older people being very talkative: ‘Because I don’t actually get that much chance to talk, so that’s why you find people like me pretty gabby’. In this way, humour functions as a way to diffuse awkwardness by smoothing over any discomfiture the story might bring to the speaker or the listener. Examining why humour might have been used, what it might reveal, and what it might hide, can assist in exploring the narratives that are often glossed over in a humorous moment of interaction.

#### Refrains

Another analytic tool is to identify the refrains, the repeated phrases that are used for narrative effect (Burck, 2005). A refrain attracts attention to a part of the story and points to the message the speaker particularly wishes to highlight. In the example on page 11–12, Jean’s refrain is ‘I’m really old’. The repeated use of this phrase in her account conveys how being old completely alters her experience of the world. Her life is no longer about plans and choices, but about pleasant fleeting interactions with the animals that visit and with nice people like Gemma. She uses this refrain as a way to make Gemma understand what it is like to live in residential care when she senses a mismatch of understanding. Jean repeats this point as it is the key message of the story she is telling, a message that she feels is being overlooked or misunderstood. By explicitly reiterating their point, the refrain points to the message the story is attempting to convey from the narrator’s perspective.

## Building a narrative analysis

Although a detailed analysis of the data is the first stage of a narrative project, narrative analysis also requires moving from the analysis of specific stories to the analysis of the data set as a whole. Not every story told or every narrative identified will be in a final analysis. To build the narrative analysis, the researcher needs to decide which stories provide answers to their research question, rather than attempting to provide a complete account of everything every participant conveyed. It is also important to recognise that different researchers will produce different analyses by influencing the research at every stage: by asking different questions, reacting differently to the participants’ stories, and finally, identifying and choosing different narratives to answer the research question.

In this research project, although the research question was initially: ‘How do older people experience the animal therapy programme?’, over the course of the research the question shifted to a focus on understanding identity in residential care through the lens of an animal therapy intervention. For example, although Cathy’s extended story on page 14–15 was not directly related to her experience of animal therapy, it did underscore some of the other narratives that related to how her age and level of frailty structured her identity, which *did* impact her experience of animal therapy. This story explained why the animal therapy programme was not significant in Cathy’s life, by pointing to the value that social interaction and conversation with people outside the facility had for Cathy. At the personal story level, this account from Cathy is a way to both justify her participation in the interview (‘to meet someone like you for example, that I can talk to you’), whilst also acknowledging that she may not be the best participant for this particular piece of research (‘So, Gemma, it’s been a bit of a lost effort for you’.). She structures this account of herself as not the right person as she is not sufficiently immobile to truly benefit from the animal therapy programme, situating herself in this moment as quite different from the residents confined to the residential care facility. This is structured by an active ageing narrative, which Gemma supports as it matches wider expectations of a good life as enjoying the outside world and leaving the care facility. This is the same narrative of a good life that underpins Gemma’s bewilderment with Jean’s satisfaction with being confined to residential care.

Building the narrative analysis requires an attention to the transcripts as a set, particularly paying attention to similarities and to differences in the stories. Do the participants tell similar stories or use the same narratives? To illustrate the process of moving from the analysis of stories to the wider narrative analysis, we describe two of the narratives from this research. One of the recurring narratives identified, exemplified by the story from Jean, was that of animal therapy as a ‘Fleeting Pleasure’. This narrative described the experience of animal therapy as brief and infrequent, but enjoyable nonetheless. For participants like Jean, bed-ridden and unable to leave residential care, the therapy was a valued part of her life. An alternative narrative that participants used was that of ‘My Life Inside and Out’. This narrative is illustrated by Andrew’s stories of his life before entering residential care and by Cathy’s stories of how she fills her days outside of residential care. In this narrative, people, activities, and events that happen outside of residential care are valued. This separates out ‘real’ life from the limited possibilities available in residential care. This narrative demonstrates the limited reach of animal therapy in altering the experience of residential care, when valued identities are firmly located outside of this context.

### A different kind of story

Although some narratives stand out because they recur across the interviews, narrative analysis is not only concerned with recurring narratives. At times, stories that do not seem to fit with the others shed light on the analysis as a whole. This links back to the way narratives are not contained in any way, and alternative accounts are often present within the stories. In this data, Annie storied her experience of animal therapy as a pure pleasure, but she also described the impact of the programme as something that ‘really does affect me’. Insight into why the impact of animal therapy was different for Annie was gained by looking at the Annie’s description of her transition into residential care:

Annie: I’d … I’d rather be in … in my home over there. Would I ever. Yeah, this is not a home; not a home-home, you know. It’s a house home. But never mind. It’s just one of those things when you get old and decrepit. (…)

Gemma: So would you say you don’t like living here?

Annie: Oh no, I wouldn’t say that at all. [Gemma: Oh right.] Oh no. [Gemma: You just prefer to be …] I’d prefer to be at my own house. This place has got a lot of people in it which is good, and a lot of them, believe it or not, used to live in [locality] and I’d see them around the … There’s just so many of them it’s incredible. Yeah. (…)

Annie: I’ve just suddenly thought of all the things I’ve given away. [Gemma: Yeah?] My sewing machine. My sewing machine, my toaster. [Gemma: Laughs] Hot water jug. You know, all these sort of things that … [sigh] are necessary. [Gemma: Are necessary?] Well, when they kick me out of here and wherever they put me, I don’t know, I’m gonna need those things.

Gemma: Is that likely that you won’t stay here?

Annie: Well, I don’t know. I don’t want to stay here forever. [Cluck]

In contrast to other participants, Annie did not imagine she would be in residential care permanently. The unique impact of the animal therapy programme on Annie was grounded in this difference. While others accepted the necessity of residential aged care as a long-term living situation, Annie saw it as a short-term solution. Because of this, she was able to appreciate positive aspects of residential care, such as the opportunities for social interaction, the quality of the staff, and the opportunity to receive visits from animals. Residential aged care structured the experiences and identities of those participants who saw it as their final chapter differently from Annie, who saw it as a temporary situation. In this way, considering both similarities and differences in the stories told strengthens the narrative analysis as a whole.

## Ethics statement

The research received ethical approval from the Massey University Human Ethics Committee.

## Conclusion

Bruner (1991) described an innovative storyteller as capable of ‘leading people to see human happenings in a fresh way, indeed in a way they had never “noticed” or even dreamed’ (p. 12). We see this as the potential of an innovative narrative analysis: to reveal the social world by presenting the analysis of stories in a novel way. This is why a prescriptive or formulaic approach to narrative analysis is insufficient; narrative analysis requires the ability to see patterns and connections as well as confusion and disjuncture to reveal the social world. The complexity of human experience, and the sometimes convoluted ways people relate their experience to others, demand flexibility and confidence to untangle. A convincing analysis requires clear and compelling signposting so the reader can see it too, once shown where to look. This paper provides some scaffolding as a way to begin, but every analysis reflects a unique combination of influences on the analysis. Familiarity with the process of analysis may help to build confidence to weave a narrative analysis out of many stories, to lead readers to see something they otherwise might have overlooked.
